# A quorum sensing-based *in vivo* expression system and its application in multivalent bacterial vaccine

**DOI:** 10.1186/s12934-015-0213-9

**Published:** 2015-03-18

**Authors:** Teng Chu, Chunshan Ni, Lingzhi Zhang, Qiyao Wang, Jingfan Xiao, Yuanxing Zhang, Qin Liu

**Affiliations:** State Key Laboratory of Bioreactor Engineering, East China University of Science and Technology, 130 Meilong Road, Shanghai, 200237 China; Shanghai Collaborative Innovation Center for Biomanufacturing, Shanghai, 200237 China

**Keywords:** Bacterial vector vaccine, *Edwardsiella tarda*, Iron uptake system, *In vivo* expression, Quorum sensing

## Abstract

**Background:**

Delivery of antigens by live bacterial carriers can elicit effective humoral and cellular responses and may be an attractive strategy for live bacterial vaccine production through introduction of a vector that expresses an exogenous protective antigen. To overcome the instability and metabolic burden associated with plasmid introduction, alternative strategies, such as the use of *in vivo-*inducible promoters, have been proposed. However, screening an ideal *in vivo*-activated promoter with high efficiency and low leak expression in a particular strain poses great challenges to many researchers.

**Results:**

In this work, we constructed an *in vivo* antigen-expressing vector suitable for *Edwardsiella tarda*, an enteric Gram-negative invasive intracellular pathogen of both animals and humans. By combining quorum sensing genes from *Vibrio fischeri* with iron uptake regulons, a synthetic binary regulation system (ironQS) for *E. tarda* was designed. *In vitro* expression assay demonstrated that the ironQS system is only initiated in the absence of Fe^2+^ in the medium when the cell density reaches its threshold. The ironQS system was further confirmed *in vivo* to present an *in vivo*-triggered and cell density-dependent expression pattern in larvae and adult zebrafish. A recombinant *E. tarda* vector vaccine candidate WED(ironQS-G) was established by introducing *gapA34,* which encodes the protective antigen glyceraldehyde-3-phosphate dehydrogenase (GAPDH) from the fish pathogen *Aeromonas hydrophila* LSA34 into ironQS system, and the immune protection afforded by this vaccine was assessed in turbot (*Scophtalmus maximus*). Most of the vaccinated fish survived under the challenge with *A. hydrophila* LSA34 (RPS = 67.0%) or *E. tarda* EIB202 (RPS = 72.3%).

**Conclusions:**

Quorum sensing system has been extensively used in various gene structures in synthetic biology as a well-functioning and population-dependent gene circuit. In this work, the *in vivo* expression system, ironQS, maintained the high expression efficiency of the quorum sensing circuit and achieved excellent expression regulation of the Fur box. The ironQS system has great potential in applications requiring *in vivo* protein expression, such as vector vaccines. Considering its high compatibility, ironQS system could function as a universal expression platform for a variety of bacterial hosts.

## Background

Vaccination constitutes the most cost-effective tool for prophylaxis of infectious diseases, and the use of bacterial carriers is probably one of the most successful strategies ever developed to deliver vaccine antigens [1]. The efficacy of a live bacterial vector vaccine rests in its ability to present sufficient antigens to the host immune system and initiate the desired protective immune response [2]. Selecting an appropriate expression strategy to optimize the production of the recombinant antigen is one of the most important issues relevant to the use of bacterial strains as vaccine carriers [3]. To date, certain strategies have been developed to achieve stable heterologous gene expression in vaccine vectors. Application of low copy number vectors and modification by introducing a balanced lethal system can improve the stability and safety of vaccines [4-6]. However, expression levels may be insufficient to stimulate the desired responses [7]. High copy number vectors achieve sufficient antigen expression but can cause over-attenuation of the carrier and lack of immunogenicity [8]. To circumvent these problems, optimal and controllable expression, such as with the use of *in vivo*-activated expression systems, is a crucial step in multivalent vaccine design.

*In vivo* environmental stimuli are represented by aggregates of unique signals, including anaerobic conditions [9], oxidizing agent availability [10], and low iron concentrations [11]. Several *in vivo*-inducible promoters that respond to these signals have been previously investigated [12]. For example, iron is often bound to metal-chelating proteins *in vivo*, making it extremely limited in hosts [13]. Thus, lack of free iron is an important *in vivo* environmental signal [14]. To survive from the iron limited conditions of their hosts, bacteria have evolved several iron uptake, storage, and metabolism systems to obtain adequate iron in such an environment [15]. The promoter from iron-uptake regulon is strongly repressed in iron-rich conditions by Fur, a typical ferric uptake regulator protein, but fully de-repressed in the absence of iron [16]. A 19-bp inverted repeat consensus sequence, Fur box, which is known as the binding site of the Fur protein complexing with ferrous irons, is the control core of iron-responsive promoters in bacteria [17]. When iron is abundant, Fur protein forms a complex with ferrous iron and blocks the entry of RNA polymerase by binding to the Fur box in the relevant promoter area [18]. In our previous work, several iron-regulated promoters and Fur boxes were applied to build *in vivo*-inducible regulation circuits for antigen expression, bacterial ghost preparation, and toxic protein synthesis [19-21].

Quorum sensing (QS) is a system that bacteria release and respond to membrane-penetrating molecules called autoinducers to regulate their gene expressions according to cell density [22]. This system allows bacteria to monitor a variety of processes, including competence, bioluminescence, virulence factor secretion, biofilm formation, and sporulation [23]. The first quorum sensing circuit was discovered in the bioluminescent marine bacterium *Vibrio fischeri* [24]. Two proteins, LuxI and LuxR, are essential for the quorum-sensing control of bioluminescence in *V. fischeri*. LuxI is the autoinducer synthase that produces an AHL, N-(3-oxohexanoyl)-homoserine lactone [25]. LuxR is the cytoplasmic receptor of the autoinducer and the transcriptional activator of the luciferase *luxICDABE* operon [26,27]. At low cell density, the *luxI* gene is transcribed at a low basal level. As the culture grows and autoinducers accumulate to a specific threshold, cytoplasmic LuxR proteins combine with autoinducers and bind to the *luxICDABE* promoters [28]. Given that the expression of *luxI* is also activated by the autoinducer-bound LuxR, this auto-induction positive feedback loop is presumed to enforce synchrony as the cell population switches from low cell density mode to high cell density quorum sensing mode [29]. Quorum sensing systems have been widely used in various gene circuits in synthetic biology, serving as oscillator [30], amplifier [31] and so on [32-34], because of their high expression efficacy and cell density regulated characteristic. However, although a number of quorum sensing systems participate in various synthetic architectures; very few have been designed for bacterial vector vaccine.

In this work, several *in vivo* expression systems, including ironQS1-4, were developed for potential application in multivalent bacterial vaccine based on quorum sensing system of *V. fischeri*. To activate the quorum sensing system *in vivo*, iron starvation of the *in vivo* environment was utilized to adopt different strategies, including iron-regulated promoter substitution and iron-regulated regulator introduction. The screened ironQS system was demonstrated to be only initiated in iron-limited medium during *in vitro* expression assay when the cell density reached a threshold. The ironQS system was also well regulated by the iron signals and cell density in the subsequent *in vivo* expression assay. Furthermore, a protective antigen, glyceraldehyde-3-phosphate dehydrogenase (GAPDH) from the important fish pathogen *Aeromonas hydrophila*, was introduced to the ironQS system. The immune protection of the resultant recombinant *E. tarda* vector vaccine candidates was evaluated in turbot.

## Results and discussion

### Construction of quorum sensing-based *in vivo* expression systems

**Figure 1:**
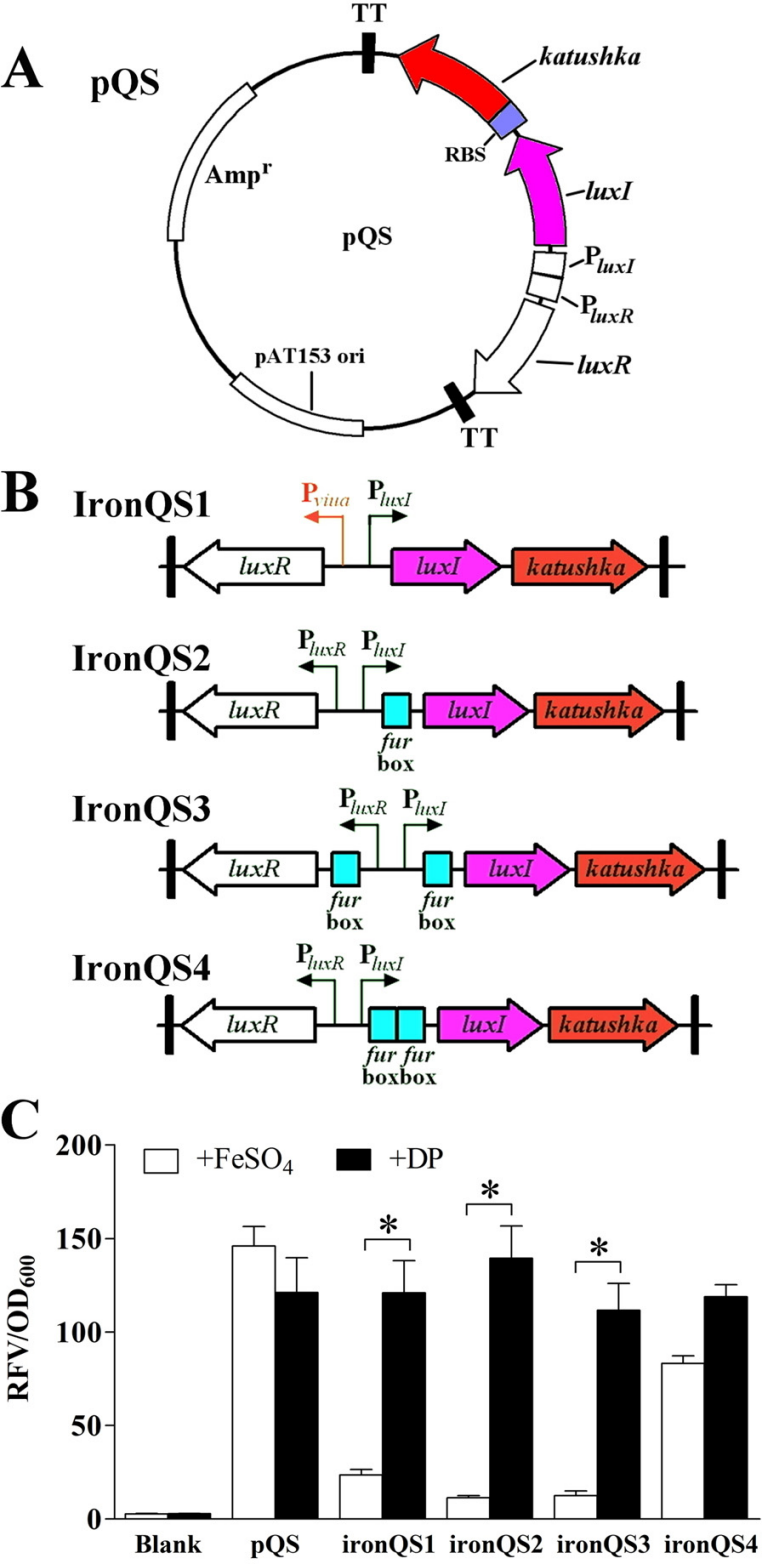
**Construction and screening of ironQS systems. (A)** Plasmid diagram of a basal cell density regulated expression plasmid pQS. P_*luxI*_, the promoter of *luxI* gene; P_*luxR*_, the promoter of *luxR* gene; TT, transcription terminator; RBS, ribosome binding site; *katushka*, a reporter gene which product generates red fluorescence. **(B)** Linear structure illustrations of four quorum sensing-based *in vivo* expression systems ironQS1-4. **(C)** Relative fluorescence values of *E. tarda* strains containing ironQS1-4 after cultured in iron-rich and iron-limited media respectively. *E. tarda* loaded with pQS and blank strain were set as controls. * means a significant deviation.

The quorum sensing components including *luxI*, *luxR* and their respective promoters were cloned from *V. fischeri*. A reporter gene named *katushka*, which encoded a far-red fluorescent protein, was used to measure the expression efficiency of our systems. First, a basal quorum sensing regulated expression circuit was constructed through sequentially inserting a RBS component and a reporter gene *katushka* behind the intact QS regulon. A stable plasmid pUTat [35] containing this circuit was named pQS (Figure 1A), and four quorum sensing-based *in vivo* expression systems ironQS1-4 were designed on this basis (Figure 1B). The ironQS1 was a pQS derivative in which the original P_*luxR*_ promoter was substituted for a strict low-iron-triggered promoter P_*viuA*_ [35], so that ironQS1 would not express Katushka protein in iron-rich culture medium. IronQS2 was constructed by inserting a standard Fur box sequence into the −10 region of *luxI* promoter, and ironQS3 and ironQS4 were the derivatives of ironQS2 with additional Fur boxes inserted striving for higher strictness. In culture medium, bacterial Fur proteins complexing with ferrous irons would bind to the Fur box sites to prevent transcriptional process of RNA polymerases, while in *in vivo* low-iron situation, ironQS2-4 systems would be de-repressed and started to produce interest proteins because of the dissociation of Fur proteins.

To evaluate the regulation and expression performances of four candidate systems, *E. tarda* recombinants harboring ironQS1-4 plasmids were cultured in both iron-limited and iron-rich media. Samples were adjusted to OD_600_ (optical density at 600 nm) = 1 to measure the red fluorescence emitted by Katushka. As seen in Figure 1C, in iron-limited environment, all four candidates showed strong fluorescence intensities with similar RFVs (relative fluorescence values) to that of the control system pQS. This was evidence that insertion of Fur box exerted no effects on expression efficiency. However, in iron-rich environment, their performances were divergent. IronQS2 presented the lowest leak expression level in iron-rich environment, while ironQS1 system showed the reduced expression strictness by the substitution of promoter P_*luxR*_ by P_*viuA*_. On the other hand, the additional Fur box in ironQS3 and ironQS4 did not play the desired effects to enhance their strictness. After comprehensive comparison of the four candidate expression systems, ironQS2, abbreviated to ironQS, was finally chosen as our desired expression system with the combined advantages of high induced expression level, high stringency and relatively simple genetic structure.

### *In vitro* expression of ironQS system

**Figure 2:**
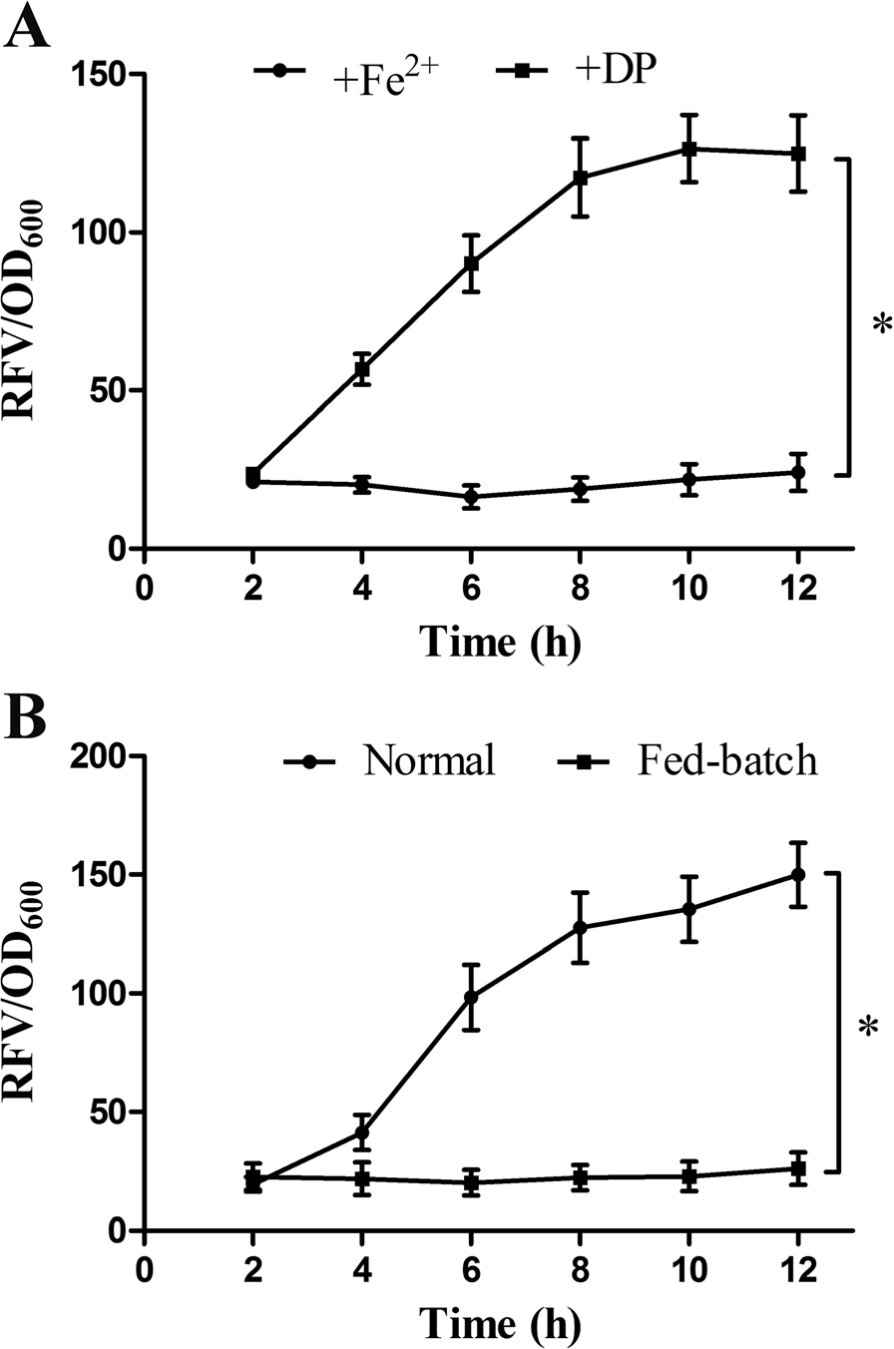
**Iron-regulated and cell density-regulated performances of ironQS system. (A)** Expression curves of ironQS system regulated by Fe^2+^ concentration. FeSO_4_ or iron-chelator DP was added into the medium during cultivation. **(B)** Expression curves of ironQS system regulated by cell density. *E. tarda* harboring ironQS system was cultivated via fed batch and the OD_600_ was maintained under 0.3 by feeding fresh iron-limited media continuously. Meanwhile a control group was carried on by cultivating the bacteria normally to a high cell density. * means a significant deviation.

To verify the iron-regulated and cell density-regulated performances of ironQS system, ironQS plasmid was transformed into *E. tarda* for the *in vitro* assay by adding FeSO_4_ or iron-chelator DP (2,2-dipyridyl) into the medium during cultivation. As showed in Figure 2A, there was a low expression level of ironQS system in the presence of Fe^2+^. This indicated that Fur protein complexing with ferrous iron bond to the Fur box region and the QS system was remarkably repressed. On the contrary, ironQS was considerably activated in the absence of iron when Fe^2+^ in the medium was plundered by DP. All these data confirmed that Fur box sequence could negatively control QS system by signaling to Fe^2+^ in the medium. Furthermore, to identify whether the cell density-regulated feature was still preserved in ironQS system, a fed-batch cultivation was conducted to control the bacterial cell density always under the threshold by continuously feeding fresh iron-free medium, and a control experiment was carried out by normally cultivating the bacteria to a high cell density. As shown in Figure 2B, RFV of ironQS strain in fed-batch model maintained at a low level, showing that the ironQS was always kept off even in iron-free medium when the cell density was below its threshold. Meanwhile, the RFV of control group rose continuously as the cell density exceeded threshold. These results suggested that ironQS system exhibited a precise iron and density dual-regulated expression feature by Fur box and quorum sensing circuit, respectively.

### *In vivo* expression of ironQS system

**Figure 3:**
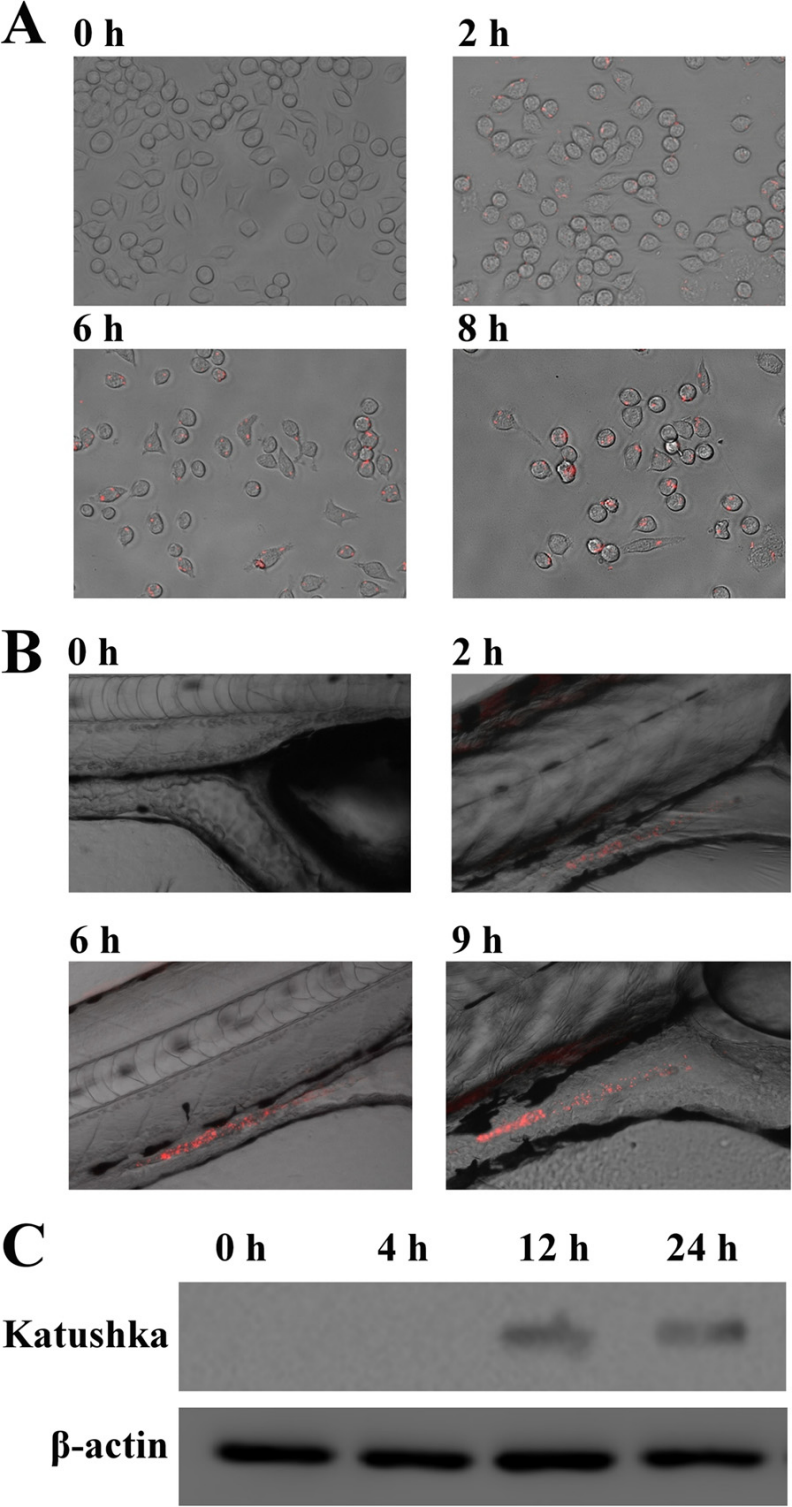
***In vivo*** **expressions of ironQS loaded** ***E. tarda*** **strains. (A)** Expression of ironQS loaded strain in macrophages J774A.1. The infected cells were observed by inverted fluorescence microscope at different time intervals. **(B)** Expression of ironQS strain in zebrafish larvae. Zebrafish larvae were immersed in *E. tarda*(ironQS) suspension and observed by inverted fluorescence microscope at different time intervals. **(C)** Expression of ironQS strain in adult zebrafish. Zebrafish were intraperitoneally injected with *E. tarda*(ironQS) suspension. The internal organs were extracted at different time intervals and further analyzed by Western blot using the antibody specific to Katushka.

*In vivo* condition is a typical iron-limited environment and a crucial site where bacterial vaccine plays its role. Although ironQS has been proved to be an efficient binary system responding to Fe^2+^ and cell density *in vitro*, the *in vivo* behaviors are particularly concerned. Since *E. tarda* is known as a facultative intracellular bacterium and able to persist and replicate within phagosomes of macrophages, it is reasonable to apply macrophages as a cell model for *in vivo* performance evaluation of ironQS. Suspensions of recombinant *E. tarda* WED containing ironQS were incubated in the macrophage-like cell J774A.1, and the fluorescence intensity was determined at regular time intervals with a fluorescence microscope. As shown in Figure 3A, cells infected by the bacterium showed only very low fluorescence in the first 2 h post-infection. During the whole period, the bacterial number maintained at 4.83 ± 1.46 per cell. Although WED was not likely to replicate in the macrophages, fluorescence intensity still became stronger gradually with the accumulation of autoinducers, indicating that ironQS system could sense the iron-limited signal in macrophage and unlock quorum sensing to express target protein. Meanwhile, the positive feedback of quorum sensing increased the expression of Katushka protein and enhanced the fluorescence intensity [36].

On the other hand, zebrafish larvae and adults were further applied as animal models to evaluate *in vivo* performance of ironQS. The larvae were infected by immersion with recombinant *E. tarda* WED strain, and the fluorescence in zebrafish larvae was determined at regular time intervals. As shown in Figure 3B, no obvious Katushka fluorescence signal was detected until 2 h post immersion and the fluorescence signals were mainly found in the gastrointestinal tract of fish. These results indicated that the ironQS system could function well in zebrafish larvae. Responding to the iron-free signal in larvae, ironQS was de-repressed after Fur protein dissociating from Fur box, and simultaneously, it was activated by locally accumulated autoinducers from an increasing bacterial population *in vivo*. For zebrafish adults, ironQS-loaded *E. tarda* strains were administrated by intraperitoneal injection, and then the *in vivo* expression pattern of ironQS was evaluated by Western blot. As showed in Figure 3C, ironQS was activated significantly after 12 h post injection upon *in vivo* propagation of the bacteria. This result indicated that attenuated *E. tarda* WED could colonize and persist in fish adult, in which the low iron concentration would trigger the expression of heterologous protein by ironQS.

All the data confirmed that ironQS system could be effectively activated *in vivo* and the bacterial density-dependent expression feature, illustrated *in vitro*, was finely preserved. IronQS system maintained its dual-regulated characteristics and made a good performance in *in vivo* environments such as in macrophage and zebrafish.

### Stress on bacterial growth and colonization

**Figure 4:**
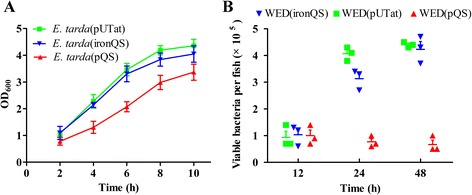
**Metabolic burden of pQS and ironQS system** ***in vitro*** **and** ***in vivo*** **. (A)** Growth curves of *E. tarda* loaded with pQS or ironQS plasmids cultivated in iron-rich medium, blank plasmid pUTat used as control. **(B)** Viable bacterial counts in each fish injected with these three strains (5 × 10^4^ CFU per tail) at 12, 24 and 48 h. Ten fish were set as a pool and three parallels were taken. At time points, 10 fish of each group were randomly picked, homogenized and the bacterial numbers were counted.

Constitutive expression systems are often believed to produce significant metabolic burden on the host bacterium and thus may have negative effects on immune protection of bacterial vector vaccine. Therefore *in vivo* inducible expression system is highly recommended for bacterial vector vaccine design [3,8]. In this work, an *in vivo* inducible and cell density-dependent expression system ironQS was established. Compared with the original QS expression system, ironQS system exerted few effects on microbial *in vitro* growth and *in vivo* colonization. As showed in Figure 4A, when bacterial hosts containing pQS, ironQS or blank plasmid pUTat were inoculated into fresh medium, WED(ironQS) showed a normal growth, which is similar to that of WED(pUTat), while the growth of WED(pQS) was obviously inhibited, especially during the early stage after inoculation. When three strains were injected into adult zebrafish, the colonization abilities were measured by counting viable bacterial numbers in zebrafish organs. As Figure 4B showed, a dramatic drop of viable bacterial numbers for WED(pQS) was found within zebrafish, while *in vivo* WED(ironQS) propagated significantly, similarly to the control strain WED(pUTat). These results suggested that the expression system ironQS, controlled by cell density and iron-limitation signal, brought about less growth stress on bacterial host and greatly contributed to the *in vivo* colonization and propagation of WED(ironQS), which would be a valuable attribute for a bacterial vector vaccine to evoke effective immune response *in vivo*.

### Expression of ironQS in broad bacterial hosts

**Figure 5:**
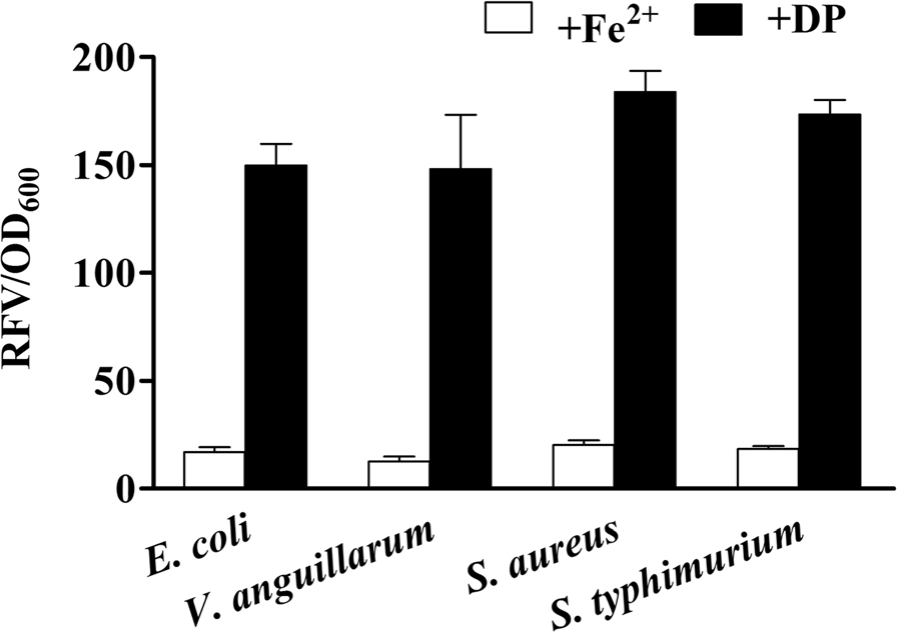
**Expressions of ironQS system in different bacterial hosts.** IronQS plasmid was respectively transformed into *Escherichia coli, Vibrio anguillarum*, *Salmonella typhimurium* and *Staphylococcus aureus* and the resultant recombinant bacteria were cultivated in LB media with addition of FeSO_4_ or DP. Cell cultures were harvested and adjusted to OD_600_ = 1 for fluorescence detection at indicated time points.

Adaptation to different bacterial hosts is an important aspect of a good heterologous expression system. IronQS system is composed of quorum sensing genes from *V. fischeri* and an artificial Fur box, and it is of paramount importance to work well in different bacterial hosts. Here, ironQS was transformed into gram-negative bacteria including genetic engineering strain *Escherichia coli*, fish-originated *Vibrio anguillarum*, and human-originated *Salmonella typhimurium*, as well as gram-positive bacterium *Staphylococcus aureus*, respectively. In *in vitro* assays, ironQS in all the four strains presented no expression in normal iron-rich medium, and high expression in DP supplemented medium (Figure 5). According to the specificity of the quorum sensing system, LuxR receptor could only sense the autoinducer molecules produced by its homologous LuxI. Thus, quorum sensing circuit from *V. fischeri* had no interference with these bacterial hosts. Fur proteins in bacterial hosts could bind to or dissociate from the standard Fur box depending on iron concentration. The fine adaptability of ironQS system suggested that it might be potential to be used in a wide range of bacterial vaccine hosts.

### Application of ironQS in multivalent bacterial vaccine

GAPDH of *A. hydrophila* has been shown to be an effective protective antigen [37]. *Edwardsiella tarda* is an important facultative intracellular pathogen of both animals and humans, and its attenuated strain WED, constructed by in-frame deleting *eseBCD* and *aroC*, is an excellent bacterial vector for use in recombinant vaccine design [38]. The ironQS vector expressing heterologous antigen GAPDH was transformed into live attenuated bacterial vaccine *E. tarda* WED, and a multivalent bacterial vaccine candidate WED(ironQS-G) was constructed.

To investigate the potential application of WED(ironQS-G) as a multivalent vaccine, protection efficacy was evaluated in turbot, an important mariculture species in China. Vaccinated turbot were maintained for 30 days and then challenged by intraperitoneal injection with wild type *A. hydrophila* LSA34 or *E. tarda* EIB202. As shown in Table 1, WED(ironQS-G) showed significant multivalent protection against *E. tarda* EIB202 (RPS = 72.3%) and *A. hydrophila* LSA34 (RPS = 67.0%) over saline control. Meanwhile, the attenuated vector strain WED triggered significant protection against *E. tarda* EIB202 (RPS = 76.7%), but only slight protection against *A. hydrophila* LSA34 (RPS = 13.9%). This confirmed that *in vivo* expression of GAPDH controlled by ironQS system conferred effective protection against experimental challenge with *A. hydrophila* LSA34, and also showed no adverse effect on the protection of *E. tarda* wildtype.

**Table 1:** **Efficacy of vaccine candidate WED(ironQS-G) against** ***E. tarda*** **EIB202 and** ***A. hydrophila*** **LSA34 in turbot**

**Immunogen**	**Vaccination dose**	**Challenge strain**	**Challenge dose (CFU/tail)**	**No. of fish**	**Mortality** ^**a**^ **(%)**	**RPS** ^**b**^ **(%)**	**Effect** ^**c**^
Saline	0.1 ml/tail	EIB202	6 × 10^3^	30 × 3^d^	100 ± 0^e^	/	/
		LSA34	8 × 10^7^	30 × 3	85 ± 5.8	/	/
WED	10^7^ CFU/tail	EIB202	6 × 10^3^	30 × 3	23.3 ± 4.7	76.7 ± 4.7	+
		LSA34	8 × 10^7^	30 × 3	73.3 ± 5.8	13.9 ± 6.8	–
WED(ironQS-G)	10^7^ CFU/tail	EIB202	6 × 10^3^	30 × 3	27.7 ± 5.0	72.3 ± 5.0	+
		LSA34	8 × 10^7^	30 × 3	28.0 ± 7.5	67.0 ± 8.9	+

^a^The mortality was recorded for 4 weeks after challenge, and the observation of surviving fish was extended to 6 weeks.

^b^RPS is the relative percent survival, which is defined relative to the saline group. RPS (%) = (1 - mortality of vaccinated fish / mortality of control fish) × 100.

^c^If RPS > 60%, it represents that the vaccine candidate showed protection against challenge, marked as “+”. If RPS < 30%, it represents no protection against challenge strain, marked as “–”.

^d^“×3” represents the vaccination and challenge experiments were done in three parallels.

^e^The “±” is standard deviation.

## Conclusions

Quorum sensing system has been widely used in various gene structures in synthetic biology as a well-functioned and population-dependent gene circuit. Although these delicate genetic architectures fully embody the density dependent features and high expression efficiency, few of such systems have been designed and implemented in practical applications [39]. In this work, an efficient and finely-regulated *in vivo* expression system, ironQS, was designed by integrating Fur box with quorum sensing circuit of *V. fischeri*. Binding of the Fur-iron complex functioned as a lock that prevents gene transcription of quorum sensing circuit and then shuts down the entire expression system. However, given the extraordinary lack of free iron *in vivo*, Fur protein dissociated from Fur box and activated quorum sensing circuit. Experiments on *in vitro* simulation and zebrafish model confirmed that the ironQS system exhibits an *in vivo*-triggered and cell density-dependent expression pattern.

For practical application, we established a bacterial vector vaccine using the ironQS system as an antigen presenter. Several characteristics dominate the efficiency of a live bacterial vector vaccine: stability, infectivity and ability to present sufficient antigen [3]. How to improve antigen delivery efficiency without reducing vaccine stability and vitality has long challenged researchers. The use of plasmids in vector vaccines is often associated with instability of the recombinant phenotype and declining ability of colonization. In the ironQS system, several strategies were adopted to render it more suitable for use in a vector vaccine. First, as the main switch of the entire system, Fur box prevented unnecessary expression during *in vitro* cultivation, thereby alleviating the metabolic burden during cultivation and enhancing the ability of colonization of the bacterial host at early infection stages [40]. Second, thorough de-repression of the quorum sensing system completely restored the high expression efficiency of the system to guarantee sufficient antigen production in the host. Benefited from the population-dependent expression pattern of quorum sensing circuit, undesirable antigen protein expression was controlled to a minimum during the first stage of colonization because of the low cell density, until the bacterial population reached its threshold and then evoked multiple protective immune responses in fish host. Taken these results together, the ironQS system proposed in this work has great potential in designs of a stable and efficient bacterial vector vaccine.

The iron-dependent gene regulation mechanism has been well studied in different bacteria, and certain iron-regulated promoters have been used to build *in vivo* inducible regulation circuits for controlled antigen expression [19,35]. Prior to designing a vector vaccine, a desirable iron-regulated promoter must be screened for the corresponding host because promoters borrowed from other host strains often function unsatisfactorily. This problem could be readily circumvented by introduction of the ironQS system. Although the various host strains used in this work possess their own QS systems and produce their own autoinducers, the LuxR receptors only identify their own autoinducers because these signal molecules vary in their acyl side chains [22,23]. Thus, no crosstalk or interference was observed between the native host QS and the heterologous ironQS because of the specificity of quorum sensing system. As well, the 19-bp consensus Fur box sequence is highly conserved [16]. Hence, the ironQS system displays high compatibility with a variety of bacterial hosts, and this plasmid forms a self-contained and closed-loop expression circuit that is less dependent on its hosts. In fact, ironQS can function as a universal platform that is suitable for multiple hosts, rather than as a specific vector for *E. tarda*, in this work. This broad suitability suggests that, theoretically, any antigen protein can be loaded into the ironQS system and this system may be introduced into any bacterial host to prepare the desired vaccines simply and directly.

In summary, we established an *in vivo* programmed expression circuit that is de-repressed by iron-free signals *in vivo* and then programmed to produce heterologous proteins in a cell density-dependent manner *in vivo*. This artificial synthetic circuit might have potential use for *in vivo* applications including, but not limited to, bacterial vector vaccines.

## Methods

### Bacterial strains and growth conditions

Strains used in this study are listed in Table 2. *E. coli* strain was grown at 37°C in Luria-Bertani (LB) medium (1% tryptone, 0.5% yeast extract, 1% NaCl). *Edwardsiella tarda*, *Aeromonas hydrophila*, *Vibrio anguillarum*, *Salmonella typhimurium* and *Staphylococcus aureus* strains were grown at 30°C in LB medium. When required, the antibiotics (Sigma, USA) ampicillin (Amp) and colistin (Col) were added at corresponding final concentrations of 100 μg/ml and 12.5 μg/ml, and FeSO_4_ (40 μM) or 2,2-dipyridyl (DP, 200 μM) was added to create iron-rich or iron-limited condition [35].

**Table 2:** **Strains and plasmids used in this study**

**Strains and plasmids**	**Information**	**Source**
Strains		
*Vibrio fischeri* MJ11	Wild type, used for cloning of *luxR/I* quorum sensing system	MCCC
*Edwardsiella tarda* EIB202	Wild type, fish pathogen, broad range testing host	Our lab
*Edwardsiella tarda* WED	Mutant disrupted in type III secretion system and chorismic acid synthesis, a live attenuated vaccine	Our lab
*Escherichia coli* BL21	General expressing strain, broad range testing host	Novagen
*Vibrio anguillarum* MVM425	Wild type, fish pathogen, broad range testing host	Our lab
*Staphylococcus aureus*	Wild type, human pathogen, broad range testing host	Our lab
*Salmonella typhimurium*	Wild type, human pathogen, broad range testing host	Our lab
*Aeromonas hydrophila* LSA34	Gene source of protective antigen GAPDH	Our lab
Plasmids		
pUTat	Expression vector, Amp^r^	Our lab [35]
pQS	pUTat vector containing intact QS gene elements and a reporter gene co-transcribed with *luxI*	This work
ironQS1	pQS plasmid derivate in which the original P_*luxR*_ promoter was substituted for a low-iron-triggered promoter P_*viuA*_	This work
ironQS2	pQS plasmid derivate inserted with a standard Fur box into −10 region of *luxI* promoter	This work
ironQS3	pQS plasmid derivate inserted with two standard Fur boxes into −10 region of *luxI* and *luxR* promoters	This work
ironQS4	pQS plasmid derivate inserted with two continuous Fur box into −10 region of *luxI* promoter	This work
ironQS-G	ironQS plasmid in which reporter gene was substituted by *gapA34* gene	This work

### Plasmid construction

General DNA operations were conducted following standard protocols. Automated DNA sequencing and primer synthesis were completed by Life Technologies (Shanghai, China). Seamless cloning operations were carried out using ClonExpress II One Step Cloning Kit (Vazyme Co. Ltd., China) following standard procedures. The plasmids used or constructed in this work were listed in Table 1. First, a basal quorum sensing regulated expression plasmid pQS was constructed on the basis of pUTat expression vector by sequentially inserting a RBS sequence and a reporter gene *katushka* behind the intact QS regulon. The DNA fragments of pUTat, *katushka* and QS gene were respectively amplified with their primers that overlapping with each other (primers pUT-F/R for linear pUTat, katushka-F/R for gene *katushka*, QS-F/R for quorum sensing circuit). By seamless cloning operation, the fragments were spliced to form the intact pQS plasmid. On the basis of pQS, four quorum sensing-based *in vivo* expression plasmids ironQS1-4 were designed by replacing promoters or inserting Fur boxes into basal plasmid pQS. IronQS1 was constructed by replacing the original *luxR* promoter with an iron-regulated promoter P_*viuA*_. P_*viuA*_ was amplified from the genome of *Vibrio anguillarum* MVM425 using primers P_*viuA*_-F/R and the rest was amplified from pQS using ironQS1-F/R. These two fractions were then jointed through seamless cloning operation. IronQS2 was constructed by inserting a standard Fur box behind *luxI* promoter to block its transcription in iron-rich condition. Using primers ironQS2-F/R, pQS plasmid was amplified, linearized and then self-ligated via seamless cloning to form ironQS2. To enhance this modification, another Fur box sequence was subsequently added behind *luxR* promoter to form ironQS3 using primers ironQS3-F/R. Similarly, in ironQS4, two continuous Fur box sequences were inserted behind *luxI* promoter for the purpose of improving iron regulation. IronQS4 was constructed by amplifying and linearizing pQS using primers ironQS4-F/R and make it self-ligated via seamless cloning operation. For construction of vaccine vector ironQS-G, the gene *gapA34* encoding glyceraldehyde-3-phosphate dehydrogenase from *Aeromonas hydrophila* LSA34 was amplified using primers gapA-F/R, and ironQS was linearized to remove the reporter gene using primers ironQSG-F/R. These two fragments were assembled together by seamless cloning operation. Primers used in this work were listed in Table 3.

**Table 3:** **Primers used in this study**

**Primers**	**Sequence (5′-3′)**
pUT-F	CAAGCTGGGTCACAGCTGAGTCGACCTGCAGCCAAGCTT
pUT-R	CCCATACTTTAAAAATTAAGGATCCCCGGGAATTC
QS-F	CCCGGGGATCCTTAATTTTTAAAGTATGGGC
QS-R	CGCCCACCATATAATTTCCTTTAATTAATTTAAGACTGC
katushka-F	TTAAAGGAAATTATATGGTGGGCGAGGATAGCGTGC
katushka-R	GGCTGCAGGTCGACTCAGCTGTGACCCAGCTTGCTC
P_*viuA*_-F	GTTTTTCATATG AATTTCTCCTTAACTCTA
P_*viuA*_-R	GATAAAGAGATGCATATGCGACTGAGCGATGTAAAAC
ironQS1-F	ATCGCTCAGTCGCATATGCATCTCTTTATCC
ironQS1-R	GAAATTCATATGAAAAACATAAATGCCGAC
ironQS2-F	GATAATGATAATCATTATCAATAAACGCAAGGGAG
ironQS2-R	GATAATGATTATCATTATCCGACTATAACAAACCATTTTC
ironQS3-F	GATAATGATAATCATTATCACCTATTGTTTGTCGC
ironQS3-R	GATAATGATTATCATTATCAAGGATAAAGAGATGC
ironQS4-F	GATAATGATAATCATTATCGATAATGATAATCATTATCAATAAACGCAAGGGAGG
ironQS4-R	GATAATGATTATCATTATCGATAATGATTATCATTATCCGACTATAACAAACCATTTTC
gapA-F	AAATTAATTAAAGGAAATTATATGACTATCAAAGTAGG
gapA-R	GCTTGGCTGCAGGTCGACTTACTTAGAGATGTGAG
ironQSG-F	CACATCTCTAAGTAAGTCGACCTGCAGCCAAGC
ironQSG-R	ACTTTGATAGTCATATAATTTCCTTTAATTAATTTAAGACTGC

### Expression assay of ironQS strains *in vitro*

Overnight bacterial cultures were inoculated (1:1000, v/v) into fresh LB medium with antibiotics and FeSO_4_. For fed-batch cultivation, fresh LB medium with antibiotics (and DP or FeSO_4_ if needed) was continuously added into culture vessel to keep OD_600_ under 0.3 all the time, below the cell density threshold to activate the established pQS system in this work. At different time intervals, each culture sample was centrifuged at 8000 × *g* for 5 min, and the harvested cells were washed with PBS for three times, and resuspended in PBS. To measure the expression level per unit of bacteria, all the samples were diluted to the same OD_600_ value (OD_600_ = 1.0) and 100 μl of each cell suspension was added into a 96-well flat-bottom polystyrene plate (Costar, USA) for fluorescence detection, using a fluorescence microplate reader (Molecular Devices SpectraMax M5, USA). For Katushka protein detection, excitation wavelength was set at 588 nm and emission at 633 nm.

### Expression assay of ironQS strains in macrophage

The mouse macrophage-like cell line J774A.1 was purchased from American Type Culture Collection (ATCC). Cells were cultured at 37°C in 24-well plate in DMEM (Gibco BRL, Eggenstein, Germany) supplemented with 10% fetal bovine serum (Gibco BRL, Eggenstein, Germany) in a humidified 5% CO_2_ atmosphere to obtain monolayers and the cell number was controlled at 3 × 10^5^ cells per well. Overnight bacterial cultures of *E. tarda*(ironQS) were inoculated (1:1000, v/v) into fresh LB medium with antibiotics and FeSO_4_, and were harvested and suspended in PBS. For each sample, 500 μl of bacterial suspension was added into the well at a moi ratio of 10:1, and incubated at 35°C for 2 h. Macrophages were then washed for three times and DMEM containing gentamicin (100 μg/ml) was added to inhibit the growth of remaining extracellular bacteria. At different time intervals, samples were observed by inverted fluorescence microscope (Leica DMI3000B, Germany). At the same time, the culture medium of corresponding wells was removed and washed with PBS for three times. Then the plate was added 500 μl Triton-100 (1%) per well and incubated in 35°C for 0.5 h. In a series of dilution, the lysed suspensions were coated on antibiotic agar plates and the bacterial number within each cell was calculated.

### Expression assay of ironQS strains in zebrafish model

Overnight bacterial cultures of *E. tarda*(ironQS) were inoculated (1:1000, v/v) into fresh LB medium with antibiotics and FeSO_4_. The bacteria were incubated for 9 h, harvested, and suspended in PBS. Zebrafish larvae at an age of the 6th to 8th day were immersed in cell suspension at a concentration of 10^8^ viable bacteria per ml for 2 h and then were washed and transferred into freshwater. At target time point, larvae were washed with PBS at least three times, anesthetized with 0.01% (w/v) anesthetic MS-222 and observed with an inverted fluorescence microscope.

For zebrafish adults, bacterial cultures were treated as above. Healthy adult zebrafish with an average weight of 0.2 g were intraperitoneally injected with *E. tarda*(ironQS) suspension at the dose of 10^5^ CFU (colony forming unit) per tail. At 4 h, 12 h and 24 h post injection, the internal organs of 20 fish from each group were surgically extracted. After homogenized and concentrated with ultrafiltration, the tissue samples were further analyzed by Western blot using the antibody specific to Katushka.

### Vaccination and challenge

The recombinant plasmid ironQS-G was transformed into attenuated *E. tarda* vaccine strain WED to get WED(ironQS-G). This potential multivalent vaccine candidate WED(ironQS-G) was assayed for the immune protection in turbot. Attenuated *E. tarda* strain WED was used as the control. All the vaccination and challenge experiments were done in triplicate. For vaccination, turbot (*Scophtalmus maximus*) weighed about 10 g from an aquaculture farm in Shandong Province, China, were fed and acclimated for 30 days before experiment. Three turbot groups were intraperitoneally injected with sterilized saline (0.1 ml per tail), WED suspension (10^7^ CFU per tail) and WED(ironQS-G) suspension (10^7^ CFU per tail), respectively. The immunized fish were reared in aquaria supplied with a continuous flow of recycling water at 16-18°C. For challenge, 4 weeks later, each immunized group was divided into 2 challenge subgroups which were intramuscularly injected with wild type *A. hydrophila* (8 × 10^7^ CFU per tail) and wild type *E. tarda* EIB202 (6 × 10^3^ CFU per tail), respectively. The mortality was recorded for 4 weeks after challenge, and the observation of surviving fish was extended to 6 weeks. The significant difference and the relative percent survival (RPS) were, respectively, calculated by using Fisher’s exact test and according to the formula:

$$ \mathrm{R}\mathrm{P}\mathrm{S}\left(\%\right)=\left(1-\mathrm{mortality}\kern0.5em \mathrm{of}\kern0.5em \mathrm{vaccinated}\kern0.5em \mathrm{fish}/\mathrm{mortality}\kern0.5em \mathrm{of}\kern0.5em \mathrm{control}\kern0.5em \mathrm{fish}\right)\times 100 $$

## Abbreviations

QS: Quorum sensing system
DP: 2,2-dipyridyl
RFV: Relative fluorescence value
GAPDH: Glyceraldehyde-3-phosphate dehydrogenase
OD: Optical density
CFU: Colony forming unit
RPS: Relative percent survival
